# Effective Control of Supraventricular Tachycardia in Neonates May Requires Combination Pharmacologic Therapy

**DOI:** 10.3390/jcm11123279

**Published:** 2022-06-08

**Authors:** Young Tae Lim, Yeo Hyang Kim, Jung Eun Kwon

**Affiliations:** 1 Department of Pediatrics, School of Medicine, Kyungpook National University, Daegu 41944, Korea; blueray14@nate.com (Y.T.L.); kimyhmd@knu.ac.kr (Y.H.K.); 2 Division of Pediatric Cardiology, Kyungpook National University Children’s Hospital, Daegu 41404, Korea

**Keywords:** newborn, arrhythmia, tachycardia, supraventricular, anti-arrhythmia agents

## Abstract

Introduction: Supraventricular tachycardia (SVT) is one of the arrhythmias that can occur in newborns. Most SVT incidents in the neonatal period are spontaneously resolved around the first year of life, but since tachycardia can frequently occur before complete resolution, appropriate medication use is required. However, no clear guidelines or consensus on the treatment of neonatal SVT have been established yet. Methods: From January 2011 to December 2021, demographic data and antiarrhythmic medications used were retrospectively analyzed for 18 newborns diagnosed with SVT at a single center. Results: A total of four medications (propranolol, amiodarone, flecainide, and atenolol) were used as maintenance therapy to prevent tachycardia recurrence, and propranolol was the most used, followed by amiodarone. Thirty-nine percent of the patients were controlled with monotherapy, but the remainder required two or more medications. The median period from medication initiation after diagnosis to the last tachycardia event was 15.5 days, and the median total duration of medication use was 362 days. None of the patients experienced any side effects of antiarrhythmic medications. The total duration of medication use was statistically significant according to the mechanism of SVT, and the usage time of the increased automaticity group was shorter than that of the re-entry group. Conclusion: Since most neonatal SVT resolves within 1 year, it is significant to provide prophylactic medication to prevent tachycardia recurrence at least until 1 year of age, and depending on the patient, the appropriate combination of medications should be identified.

## 1. Introduction

Supraventricular tachycardia (SVT) is a tachycardia that originates from the atria or atrioventricular (AV) node and appears as a narrow QRS complex tachycardia in the electrocardiogram (ECG). The first peak incidence of SVT onset is during the neonatal period, and most of them resolve spontaneously after the patient reaches 1 year of age [1,2]. Neonates cannot complain of tachycardia and express only sagging, nausea, and vomiting; therefore, it is necessary to manage SVT that occurs at this time to prevent recurrence through prophylactic antiarrhythmic drug treatment until resolution.

To date, consensus on guidelines for drug treatment has not been established for SVT that occurs during the neonatal period; thus, treatment drugs are selected according to the doctor’s experience or preference. Traditionally, propranolol and digoxin were the most commonly used antiarrhythmic drugs [3]; recently, class III antiarrhythmic drugs (sotalol and amiodarone) and class IC drugs (flecainide and propafenone) have been widely used [4].

This study suggests a treatment direction for newborns with SVT by evaluating the types, doses, and remission periods of drugs used in patients diagnosed with SVT in the neonatal period at a single tertiary medical center.

## 2. Methods

### 2.1. Patients

This study was conducted on patients diagnosed with SVT in the neonatal period at the department of pediatrics in a tertiary medical center (Kyungpook National University Children’s Hospital, Daegu, Korea) from January 2011 to December 2021. The electronic medical records of patients included in the study were analyzed retrospectively. Patients with tachycardia after heart surgery or those accompanied by complex heart disease were excluded.

The diagnosis of arrhythmia was evaluated by 12-lead ECG in tachycardia and at normal pulse. The symptoms at the time of diagnosis were categorized as follows: (1) shock state, (2) heart failure state, (3) complaining of symptoms without shock or heart failure, and (4) incidental finding.

All the patients underwent echocardiography to differentiate arrhythmia from accompanying congenital heart disease. Data on gender, age at the time of diagnosis, initial symptoms at the time of diagnosis, weight at the time of diagnosis, and whether the infant was premature were investigated.

### 2.2. Antiarrhythmic Medication

Adenosine, DC cardioversion, and intravenous amiodarone and esmolol were used as acute therapy to control tachycardia. To prevent recurrence after the stabilization of tachycardia, propranolol, atenolol, amiodarone, and flecainide were used.

In our center, propranolol was used first as a prophylactic medication. If tachycardia recurrence occurred even though propranolol was used, the propranolol dose was increased to the capacity to be used for the patient’s weight, or other antiarrhythmic drugs were added, such as amiodarone and flecainide. If there was a concern about the side effects of amiodarone, the use of flecainide was considered, and in other cases, amiodarone was used first. If the recurrence of tachycardia was not controlled even though two drugs were used in combination, a third drug was added, and if either amiodarone or flecainide were unused, it was added. Atenolol was used as an alternative to propranolol when tachycardia was not controlled well despite the use of the maximum dose of propranolol or if compliance with propranolol administration (4 times a day) was poor. The prophylactic medications used after the last tachycardia event were defined as “final maintenance therapy”.

The type and number of prophylactic antiarrhythmic medications used, the period from the first initiation of the drug to the last tachycardia event, and the total duration of medication were investigated in all patients. Additionally, patients were divided into two groups according to the mechanism of SVT, and the groups were compared.

### 2.3. Statistical Analysis

Statistical analysis was performed using IBM SPSS, version 26. Continuous variables were expressed as median and interquartile range (25th–75th percentile), and nominal variables were expressed as percentages. The Mann-Whitney test was used to compare the two groups divided according to the mechanism of SVT. A *p* value of <0.05 was considered statistically significant.

### 2.4. Ethics Statement

This study was reviewed and approved by the Institutional Review Board of Kyungpook National University Chilgok Hospital (IRB file no. 2022-03-014) and the Institutional Review Board of Kyungpook National University Hospital (IRB file no. 2022-03-029).

## 3. Result

### 3.1. Patients’ Characteristics

A total of 18 patients (males, 13 (72.2%); females, 5 (27.8%)) were included in the study. The median age at the time of diagnosis was 10.5 (interquartile range (IQR), 7.25–19.8 days). The median weight and age at birth were 3.66 (IQR, 3.49–4) kg and 38.5 (IQR, 37.8–40) weeks, respectively, and there were two preterm infants (Table 1).

At the time of diagnosis, tachycardia was incidentally found in nine (50%) patients. Moreover, heart failure was found in four (22.2%) patients, and only minor symptoms, such as irritability, were found in three (16.7%) patients. Two (11.1%) children were hospitalized in a shock state. The mechanism of tachycardia was determined by ECG during tachycardia and normal pulse. Twelve patients were diagnosed with paroxysmal SVT due to re-entry, and six patients were diagnosed with five episodes of atrial tachycardia and one episode of junctional tachycardia due to increased automaticity (Table 2).

### 3.2. Antiarrhythmic Medication

Adenosine, an acute therapy, was used in 15 (83%) patients and was effective in 11 (73%) patients. Intravenous amiodarone and esmolol were used in nine (60%) and five (28%) patients, respectively.

Among prophylactic medications, propranolol was the most frequently used medication (18/18, 100%), followed by amiodarone in 10 (56%) patients. Flecainide and atenolol were used in seven (39%) and four (22%) patients, respectively.

Seven (39%) patients used only one medication as maintenance therapy, and all of them used propranolol alone. A combination of two medications was used in five (28%) patients, including propranolol + amiodarone in four and propranolol + flecainide in one. Five (28%) patients used a combination of three medications, including propranolol + amiodarone + flecainide in two, atenolol + amiodarone + flecainide in two, and propranolol + atenolol + amiodarone in one. One patient used all four drugs simultaneously (Table 3).

The median period from medication initiation after diagnosis to the last tachycardia event was 15.5 (IQR: 5.3–29.3) days. The median total duration of medication use was 362 (IQR: 223–476) days in 14 patients, excluding 2 who were still taking medications and another 2 who were lost during follow-up due to personal reasons while taking medications (Table 2).

No patients developed drug side effects. The median hospitalization period was 26.5 (IQR: 8.25–38.3) days, and no hospitalization cases due to tachycardia recurrence after discharge were noted.

### 3.3. Re-Entry vs. Increased Automaticity

All patients were divided into a re-entry group or an increased automaticity group according to the mechanism of tachycardia, and their sizes were compared. The re-entry group was older at diagnosis (median, 17.5 vs. 7.5 days, *p* = 0.049) and had a lower birth weight (median, 3.22 vs. 3.77 kg, *p* = 0.022), which showed statistically significant size differences. The number of prophylactic medications used was higher in the re-entry group, but there was no statistical difference. The period from medication initiation after diagnosis to the last tachycardia event was also longer in the re-entry group but not statistically significant, whereas the total duration of medication use was significantly longer in the re-entry group compared with the automaticity group (median 444.5 vs. 196.5 days, *p* = 0.02). In the case of SVT caused by increased automaticity mechanism, there were no cases of using three or more prophylactic medications. In 50% of patients, tachycardia was controlled by propranolol alone, and in the other half, tachycardia was controlled by the combination of propranolol and amiodarone. On the other hand, in the re-entry group, tachycardia was controlled by propranolol alone in some cases but all four medications in other cases (Table 4).

## 4. Discussion

SVT is defined as a narrow QRS complex tachycardia that requires atrial tissue or AV node as an arrhythmia substrate [1], and the mechanism of development is divided into re-entry and automaticity [5,6]. In children, SVT is mainly caused by the re-entry mechanism; however, it is known that SVT due to the automaticity mechanism is more prevalent in infants than that in children or adolescents and accounts for 15% of total SVT incidents [7]. In this study, re-entry and automatic tachycardia occurred in 67% and 33% of the patients, respectively, which was consistent with the results of other studies.

For SVT in adolescents and adults, radiofrequency catheter ablation is recommended for treatment [8]. However, if ablation is performed in children <4 years of age or weighing <15 kg, adjacent structures such as AV nodes and coronary arteries may be damaged. Since most SVTs in newborns have a spontaneous resolution, the main treatment is to take prophylactic antiarrhythmic medications to prevent tachycardia recurrence [4].

Propranolol, a class II antiarrhythmic agent used for various purposes, e.g., as an antianginal agent and an antihypertensive medication, is a nonselective beta-adrenergic blocker that lowers the heart rate. A previous study conducted on 2848 infants diagnosed with SVT showed significant practice variation in the secondary prevention of SVT [9]. Cardiac glycosides such as digoxin and beta blockers were the most frequently used prevention therapies for SVT. The frequency of digoxin use gradually decreased, whereas beta-blocker use gradually increased. In the present study, propranolol was the most commonly used drug, and digoxin was not used at all. Propranolol was used as the first drug in all patients diagnosed with SVT, regardless of the mechanism of occurrence, and was included in the maintenance therapy in all patients except for two.

Atenolol is a second-generation beta-1 adrenergic antagonist that inhibits sympathetic stimulation by blocking the positive inotropic and chronotropic actions of endogenous catecholamines. Consequently, the heart rate decreases, and the refractory period of the AV node increases [10]. Atenolol is a long-acting beta-adrenergic antagonist; therefore, it has a longer half-life than propranolol. It has the advantage of reducing the number of medications taken per day. In a previous study of patients with AVRT < 5 years old examining the long-term efficiency of atenolol, arrhythmia was controlled in approximately 70% of the patients, with no specific side effects observed in all patients using atenolol [11]. In the present study, atenolol was used as maintenance therapy in four patients with re-entry tachycardia. Since the participants of the present study were newborns, propranolol was used first; if arrhythmia was not controlled, atenolol was used as an additional or alternative drug. No side effects, such as bradycardia, were noted. Considering the advantages, such as the number of doses, atenolol can be considered a prophylactic medication.

In the present study, if an additional drug was needed because heart rate control was not achieved with propranolol monotherapy, amiodarone was added first. Amiodarone is a class III antiarrhythmic agent and is highly effective in treating SVT; however, it is associated with adverse effects involving several organs. Particularly in children since proarrhythmia, keratopathy, abnormal thyroid function, and hepatitis may occur as side effects [12], caution should be observed while using amiodarone. According to several reports on the use of amiodarone in neonates and infants, amiodarone can be an effective and safe treatment for tachycardia if the side effects can be monitored well [13,14,15]. In the present study, no side effects were observed in patients using amiodarone.

Flecainide is a class IC antiarrhythmic agent with the effect of slowing conduction in a wide range of cardiac tissues and prolonging the refractory period [16]. Recently, it has been reported that flecainide is widely used as an SVT prophylactic medication for infants [4]. In a previous study that analyzed the pharmacological treatment of infant SVT at a single center for 24 years, flecainide was used as the first therapy to prevent tachycardia recurrence in re-entry tachycardia. It was effective in 97% of patients and had few serious adverse reactions, making it safe for use in infants [17]. In the present study, flecainide was added as the third drug when heart rate control was not achieved with the combination therapy of propranolol and amiodarone, mainly in re-entry tachycardia, and all of them achieved heart rate control. Considering these results, flecainide can be considered the first choice rather than an alternative therapy for tachycardia recurrence.

In the mature heart, the AV node only shows myocardial continuity between the atria and ventricles, and the rest is separated by fibrous tissue at the AV junction. However, in some newborns, the fibrous tissue between the atria and ventricle is still less formed or less mature at birth; therefore, accessory pathways may exist in addition to the AV node, which may lead to conduction from the atria to the ventricle. The annulus fibrosus between the atrium and ventricles matures during the first year of life, and accessory pathways spontaneously disappear. This explains the spontaneous disappearance of SVT after patients diagnosed with SVT during the neonatal and infantile periods reach 1 year of age [18]. In this study, no patient with tachycardia recurrence after discharge was noted. It is believed that SVT spontaneously resolved because prophylactic medication reduced the possibility of tachycardia during the infantile period and cardiac maturation continued after birth.

The participants of this study were able to gradually reduce the drug dose after discharge and discontinued drug usage at approximately 12 months of age without tachycardia recurrence. This is consistent with the time when SVT spontaneously resolves through the infantile period. However, in a study by Osman et al., the recurrence rate was compared in infants with SVT with different durations of prophylactic medication, and no difference in the recurrence rate was observed among groups treated with drugs for <6, 6–12, or >12 months [19]. Considering these results, if the patient is doing well without tachycardia, it may be possible to plan the duration of prophylactic medication to be shorter than the results in the present study.

Similar to the spontaneous resolution of the accessory pathway in re-entry-type SVT, it can be considered that the focus of abnormal cells in automatic-type SVT and with more rapid phase 4 depolarization, which causes tachycardia, may have naturally resolved over time. In a study investigating whether the clinical course of atrial ectopic tachycardia is age dependent, it was found that the spontaneous resolution of arrhythmia was significantly better in the group under 3 years of age compared with those age 3 or older [20]. According to the results of the present study, when the mechanism of SVT was increased automatically, the number of prophylactic medications used was smaller than that of re-entry-type SVT, and the total duration of taking medication was shorter. It can be speculated that the abnormal focus of automatic-type SVT has a faster spontaneous resolution than the accessory pathway of re-entry-type SVT. Therefore, identifying exactly what the mechanism of SVT is at the time of initial SVT diagnosis will also help in predicting the treatment process in the future.

This study has some limitations. First, this study had a small sample size. Second, this study had a single-center and retrospective design. However, it was possible to discover which treatment was used and effective in a situation where guidelines or consensus on treatment were not established in neonatal SVTs.

## 5. Conclusions

Since most neonatal SVT resolves within 1 year, it is important to provide adequate prophylactic medication to prevent tachycardia recurrence until at least 1 year of age. Available medications include propranolol, atenolol, amiodarone, and flecainide. In addition, accurate identification of the mechanism of SVT at the time of initial diagnosis may help treat SVT.

## Figures and Tables

**Table 1:** Patient characteristics (continuous variables).

Characteristics	Study Participants (*n* = 18)
Age at diagnosis (days)	10.5 (7.25, 19.8)
Weight (kg)	3.66 (3.49, 4)
Gestational age (weeks)	38.5 (37.8, 40)
The period from starting medication to the last tachycardia event (days)	15.5 (5.3, 29.3)
The total duration of medication use (days)	362 (223, 476)

**Table 2:** Patient characteristics (nominal variables).

Characteristics	Study Participants (*n* = 18)
Sex	
Male (*n*, %)	13 (72.2)
Mechanism	
Re-entry (*n*, %)	12 (66.7)
Automaticity (*n*, %)	6 (33.3)
Initial symptoms	
Incidental (*n*, %)	9 (50.0)
Heart failure (*n*. %)	4 (22.2)
Shock (*n*, %)	2 (11.1)
Minor (*n*, %)	3 (16.7)
Number of drugs used (maintenance therapy)	
1 (*n*, %)	7 (38.8)
2 (*n*, %)	5 (27.8)
3 (*n*, %)	5 (27.8)
4 (*n*, %)	1(5.6)

**Table 3:** Type of supraventricular tachycardia and medication.

Patients	Type	Onset (Days)	Time to CV (Days)	Final Maintenance Therapy	Total Medication Duration (Days)
1	Re-entry	35	30	PR + AM + AT	In treatment
2	Re-entry	20	21	PR + AM + FL	322
3	Re-entry	28	16	PR + AM + FL	507
4	Re-entry	35	0	PR	574
5	Re-entry	9	54	PR + AM	1212
6	Re-entry	8	42	AM + FL + AT	1227
7	Re-entry	19	126	AM + FL + AT	F/U loss
8	Re-entry	16	170	PR + AM + FL + AT	In treatment
9	Re-entry	4	27	PR + FL	384
10	Re-entry	11	6	PR	F/U loss
11	Re-entry	6	11	PR	272
12	Re-entry	33	0	PR	350
13	Automatic	7	5	PR	206
14	Automatic	4	13	PR + AM	373
15	Automatic	10	15	PR + AM	187
16	Automatic	14	23	PR + AM	380
17	Automatic	5	1	PR	55
18	Automatic	8	1	PR	109

CV = conversion to sinus rhythm; PR = propranolol; AM = amiodarone; AT = atenolol; FL = flecainide; F/U = follow-up.

**Table 4:** Comparison of the two groups according to SVT mechanism.

Characteristics	Re-Entry (*n* = 12)	Automaticity (*n* = 6)	*p*-Value
Age at diagnosis (days)	17.5 (8.25, 31.75)	7.5 (4.75, 11)	0.049
Weight (kg)	3.22 (2.73, 3.54)	3.77 (3.21, 4.22)	0.022
Gestational age (weeks)	38.35 (37.4, 39.5)	39.3 (38.1, 40.6)	0.26
Number of drugs used	2.5 (1, 3)	1.5 (1, 2)	0.139
The period from starting medication to the last tachycardia event (days)	24 (7.25, 51)	9 (1, 17)	0.134
The total duration of medication use (days)	445.5 (329, 1052.5)	196.5 (95.5, 374.8)	0.02

## Data Availability

The data presented in this study are available on request from the corresponding author.
